# Expression of the lncRNA Maternally Expressed Gene 3 (MEG3) Contributes to the Control of Lung Cancer Cell Proliferation by the Rb Pathway

**DOI:** 10.1371/journal.pone.0166363

**Published:** 2016-11-10

**Authors:** Traci L. Kruer, Susan M. Dougherty, Lindsey Reynolds, Elizabeth Long, Tanya de Silva, William W. Lockwood, Brian F. Clem

**Affiliations:** 1 Department of Biochemistry and Molecular Genetics, James Graham Brown Cancer Center, University of Louisville, Louisville, Kentucky, United States of America; 2 Department of Pathology and Laboratory Medicine, University of British Columbia, Vancouver, BC, Canada; 3 Integrative Oncology, BC Cancer Research Centre, Vancouver, BC, Canada; University of South Alabama Mitchell Cancer Institute, UNITED STATES

## Abstract

Maternally expressed gene 3 (*MEG3*, mouse homolog *Gtl2*) encodes a long noncoding RNA (lncRNA) that is expressed in many normal tissues, but is suppressed in various cancer cell lines and tumors, suggesting it plays a functional role as a tumor suppressor. Hypermethylation has been shown to contribute to this loss of expression. We now demonstrate that MEG3 expression is regulated by the retinoblastoma protein (Rb) pathway and correlates with a change in cell proliferation. Microarray analysis of mouse embryonic fibroblasts (MEFs) isolated from mice with genetic deletion of all three Rb family members (TKO) revealed a significant silencing of Gtl2/MEG3 expression compared to WT MEFs, and re-expression of Gtl2/MEG3 caused decrease in cell proliferation and increased apoptosis. MEG3 levels also were suppressed in A549 lung cancer cells compared with normal human bronchial epithelial (NHBE) cells, and, similar to the TKO cells, re-constitution of MEG3 led to a decrease in cell proliferation and elevated apoptosis. Activation of pRb by treatment of A549 and SK-MES-1 cells with palbociclib, a CDK4/6 inhibitor, increased the expression of MEG3 in a dose-dependent manner, while knockdown of pRb/p107 attenuated this effect. In addition, expression of phosphorylation-deficient mutant of pRb increased MEG3 levels in both lung cancer cell types. Treatment of these cells with palbociclib also decreased the expression of pRb-regulated DNA methyltransferase 1 (DNMT1), while conversely, knockdown of DNMT1 resulted in increased expression of MEG3. As gene methylation has been suggested for MEG3 regulation, we found that palbociclib resulted in decreased methylation of the MEG3 locus similar to that observed with 5-aza-deoxycytidine. Anti-sense oligonucleotide silencing of drug-induced MEG3 expression in A549 and SK-MES-1 cells partially rescued the palbociclib-mediated decrease in cell proliferation, while analysis of the TCGA database revealed decreased MEG3 expression in human lung tumors harboring a disrupted RB pathway. Together, these data suggest that disruption of the pRb-DNMT1 pathway leads to a decrease in MEG3 expression, thereby contributing to the pro-proliferative state of certain cancer cells.

## Introduction

The retinoblastoma tumor suppressor (pRb) pathway is disrupted in many human cancers. pRb regulates cell cycle progression and proliferation by binding to the E2F family of transcription factors and repressing their activity. For cell cycle progression to occur, pRb must be inactivated through phosphorylation by cyclin dependent kinases (CDKs), specifically CDK4 and CDK6 [1]. This phosphorylation causes pRb to dissociate from the E2F transcription factors, allowing transcription of genes required for S-phase and G2/M progression.

While the Rb pathway is a major driver controlling cell division, other mechanisms also function to regulate cell cycle progression and cell proliferation, including downstream effectors of certain miRNAs and long non-coding RNAs (lncRNAs). Specifically, the lncRNA maternally expressed gene 3 (MEG3) has been shown to function as a tumor suppressor and inhibit cell proliferation in a number of human cancer cell lines [2–7]. Although the mechanism for this is unclear, MEG3 has been shown to inhibit cell proliferation by both activation of p53, and by targeting the TGF-β pathway through direct chromatin interactions [3, 7, 8].

MEG3 is a maternally imprinted gene located on chromosome 14q32.3 and was first identified as its mouse homologue Gtl2 [9]. Imprinting of this gene is controlled by the upstream intergenic differentially methylated region (IG-DMR) [10]. MEG3 is expressed in a variety or normal tissues, however the expression has been lost in many human cancer cell lines and tumors (reviewed in [11]. Hypermethylation of both the MEG3 promoter and the IG-DMR have been shown to lead to loss of expression in human cancer cells [12, 13]. Previous studies have shown that inhibition or knockdown of DNA methyltransferase 1 (DNMT1) can restore MEG3 expression in human hepatocellular carcinoma cells and hepatic stellate cells [2, 14, 15]. The pRb/E2F1 pathway has previously been shown to negatively regulate DNMT1 and inactivation of this pathway led to hypermethylation and silencing of certain tumor suppressor genes [16].

In this study, we demonstrate a novel mechanism of the regulation of MEG3 expression by pRb through DNMT1. We show that activation of pRb by treatment of lung carcinoma cells with palbociclib, a CDK4/6 inhibitor, increased the expression of MEG3 in a pRb-specific manner. Importantly, silencing of drug-induced MEG3 expression partially rescued the palbociclib-mediated decrease in cell proliferation and analysis of human lung tumors revealed a decrease in MEG3 expression in cells harboring genetic alterations known to inactive the RB pathway. These data suggest that MEG3 is a downstream effector of the Rb pathway in controlling lung cancer proliferation.

## Materials and Methods

### Cell culture

TKO MEFs were originally provided by Julian Sage [17] and the A549 and SK-MES-1 lung cancer cells were obtained from ATCC. MEFs and A549 cells were cultured in DMEM (Gibco) supplemented with 10% fetal bovine serum (Hyclone) and 50 μg/ml gentamicin sulfate (Gibco). SK-MES-1 cells were cultured in RPMI-1640 (Gibco) supplemented with 10% fetal bovine serum (Hyclone) and 50 μg/ml gentamicin sulfate (Gibco). Palbociclib (PD0332991) was purchased from Selleck Chemicals. Cell number and viability was determined by trypan blue exclusion and enumeration using a hemocytometer.

### Microarray

In triplicate, 100 ng of total RNA from either WT or TKO MEFs was amplified and labeled following the Affymetrix (Santa Clara, CA) standard protocol for their 3’IVT Express Labeling Kit, followed by hybridization to Affymetrix MOE430 2.0 Expression arrays. The arrays were processed following the manufacturer recommended wash and stain protocol on an Affymetrix FS-450 fluidics station and scanned on an Affymetrix GeneChip^®^ 7G scanner using Command Console 3.1. The resulting.cel files were imported into Partek Genomics Suite 6.5 and transcripts were normalized and summarized using RMA as normalization and background correction method. Contrasts in a 1-way ANOVA were set up to compare the groups of interest. FDR was chosen as multiple test correction for the resulting p-values.

### Cell transfections

Cells were transfected with a plasmid encoding either mouse *Gtl2* (Origene), human *MEG3* (Origene), PSM-Rb or empty vector using Lipofectamine 2000 (Life Technologies). pQCXIH-PSM-Rb was a gift from Joseph Nevins (Addgene plasmid # 37106) and was sub-cloned into pcDNA3.1[18]. siRNA transfections were performed using RNAiMAX (Life Technologies) with the following siRNAs at a concentration of 45 nM: RB1 (Ambion: s522), p107 (RBL1) (Ambion: s11853), DNMT1 (Ambion: s4215). For MEG3 knockdown, cells were transfected with 10 nM control LNA GapmeR antisense oligonucleotide (ASO) or MEG3 LNA GapmeR ASO (Exiqon) using Lipofectamine RNAiMAX (Life Technologies) and allowed to incubate for 24 h prior to palbociclib treatment. The target sequence for MEG3 was as follows: 5’-GTAAGACAAGCAAGAG-3’.

### Real-time PCR

Total RNA was obtained using the RNeasy kit (Qiagen). The resulting total RNA (1 μg) was reverse transcribed using the High Capacity RNA-to-cDNA Kit (ABI). Gene expression was determined using specific Taqman Gene Expression Assays with the following probes: MEG3-HS00292028_m1, RB1-Hs01078066_m1, RBL1-Hs00765700_m1, DNMT1-Hs00945875_m1, β-actin-Hs01060665_g1 (ABI). β-actin was used as an internal control.

### Western Blotting

Western blot analysis was performed on protein lysates from A549 and SK-MES-1 cells using antibodies against phospho-Rb (S780, Cell Signaling), total Rb (4H1, Cell Signaling)or p107 (13354-1-AP, Proteintech). PSM-Rb was detected using Rb antibody C-15 (Santa Cruz).

### Cell Cycle, Apoptosis and BRDU Analysis

For cell cycle analysis, cells were washed in PBS and fixed in 70% ethanol for 24 h at -20°C. After fixation, cells were washed with PBS and stained with 50 μg/ml propidium iodide (PI) containing 200 mg/ml RNase A at 37°C for 30 min. in the dark and analyzed by flow cytometry. Apoptosis was measured using the FITC Annexin V Apoptosis Detection Kit I (BD Pharmingen) and measured by flow cytometry. Cell proliferation was measured by BrdU incorporation using a BrdU Flow Kit (BD Pharmingen) according to the manufacturer’s instructions. Cells were analyzed by flow cytometry using a FACSCalibur (BD Biosciences) and data analyzed using FloJo (TreeStar).

### Methylation-Specific PCR (MSP)

Cells were treated with either 1 μM 5-aza-2’-deoxycytidine (5-azadC) (Sigma-Aldrich) or 1 μM palbociclib for 48 h. Genomic DNA was extracted using the QIAamp DNA mini kit (Qiagen) and bisulphite treatment was performed using the EZ DNA methylation kit (Zymo Research). The methylation status of MEG3 was determined by MSP as previously described [19].

### Genomic analyses of human lung adenocarcinomas

Genomic data for 230 lung adenocarcinomas from The Cancer Genome Atlas was downloaded from the cBIO Portal (PMID: 23550210 & 22588877) including mutation (from whole exome-sequencing), putative copy-number alteration (from GISTIC) and mRNA Expression (RSEM and z-Scores from RNA-Seq) for *RB1*, *CDKN2A*, *CCND1* and *MEG3*. Genes status was the determined as mutated (non-silent coding mutation), homozygously deleted (GISTIC value <-2) or over/underexpressed (RNA-seq z-score >2 or <-2 respectively. The different dimensions of data were for each gene were then aligned and the RB pathway was considered disrupted in a tumour if one of the following events occurred: 1) RB1 mutation/homozygous deletion/underexpression; 2) CDKN2A mutation/homozygous deletion/underexpression; or 3) CCND1 overexpression. *MEG3* expression (RNA-seq RSEM values) were then plotted along with the disruption status of all genes and the RB pathway using GENE-E software (http://www.broadinstitute.org/cancer/software/GENE-E/index.html) and the expression of *MEG3* between the RB disrupted and non-disrupted groups was compared using an unpaired t test with Welch’s correction and plotted using Prism Graphpad software.

### Statistical Analysis

Statistical significance between the means of two experimental groups (empty vector versus Gtl2/MEG3, vehicle versus palbociclib, control versus PSM, or control versus specific siRNA) for cell number, cell cycle analysis, apoptosis, real-time PCR measurements, phospho/total RB expression, and BrdU incorporation was determined by two-tailed student t-test using Graphpad Prism. *P* ≤ 0.05 was considered statistically significant.

## Results

### Gtl2 is down-regulated in Rb-family triple knock-out (TKO) MEF cells and re-expression suppresses proliferation and increases apoptosis

**Fig 1:**
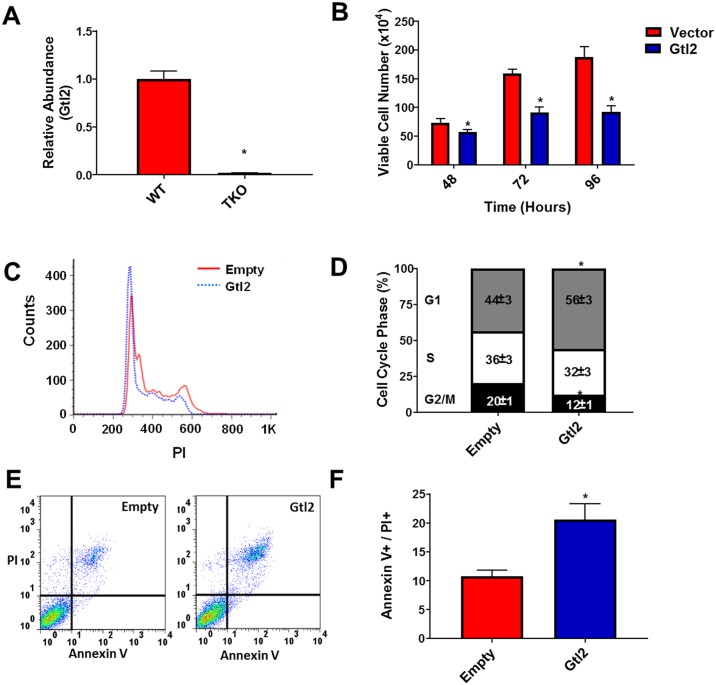
Gtl2 is down-regulated in Rb-family triple knock-out (TKO) MEF cells and re-expression inhibits proliferation and increases apoptosis. (A) Relative expression of Gtl2 was determined by qPCR in WT and TKO MEFs. (B) TKO MEFs were transfected with either a plasmid encoding mouse Gtl2 or empty vector and viable cell number was determined at 48, 72 and 96 h. Results are shown as mean ± S.D. for results from at least three independent experiments. (C&D) Distribution of the cells in the G1, S and G2/M phases of the cell cycle after 48 h by propidium iodide (PI) staining and flow cytometry. (E&F) Apoptotic rates were measured by the presence of PI and Annexin V positive cells after 48 h by flow cytometry. *p<0.05.

Microarray analysis comparing WT mouse embryonic fibroblasts (MEFs) and MEFs isolated from mice genetically deleted of all three Rb family members (Rb-1, Rbl1 and Rbl2) [TKO] revealed that Gtl2 expression is significantly decreased in TKO MEFs compared to WT MEFs (76-fold decrease, p = 4x10^-13^). These results were subsequently confirmed through qPCR analysis of Gtl2 expression (Fig 1A). To determine the effect of Gtl2 re-expression on cell proliferation, TKO MEFs were transfected with either a plasmid encoding mouse Gtl2 or empty vector and viable cell number was determined at 48, 72 and 96 hours. Reconstitution of Gtl2 in the TKO MEFs (S1 Fig) significantly decreased proliferation at each time point compared to control (Fig 1B). To examine the effect of Gtl2 on cell cycle progression in TKO MEFs, propidium iodide staining was analyzed by flow cytometry (Fig 1C & 1D). Cells overexpressing Gtl2 showed an increase in the G1 phase and a decrease in G2/M. To determine if apoptosis also contributed to the decrease in cell number, the apoptotic rate of TKO MEFs transfected with either a plasmid encoding mouse Gtl2 or empty vector was measure by flow cytometry (Fig 1E & 1F). Cells transfected with Gtl2 showed an increase in apoptosis compared to cells transfected with empty vector.

### Inactivation of Rb leads to decreased expression of MEG3 and re-expression inhibits proliferation in human lung adenocarcinoma cells

**Fig 2:**
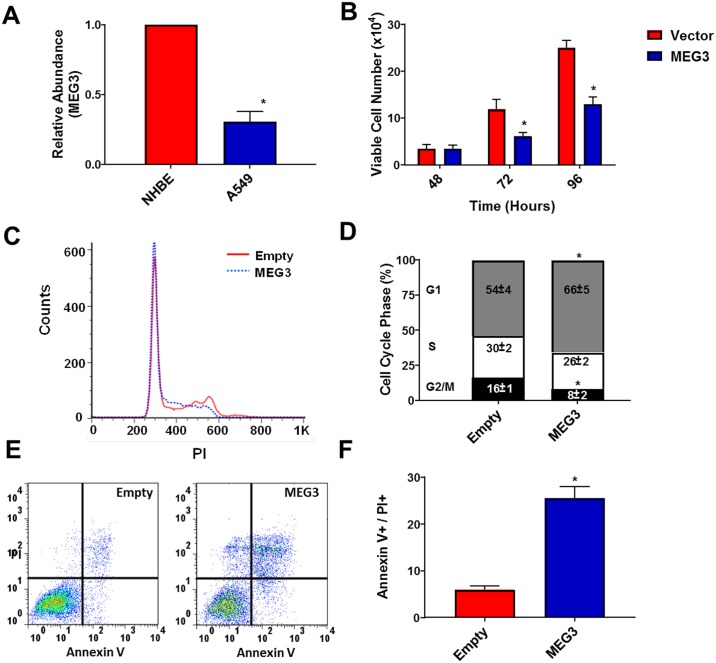
Inactivation of Rb leads to decreased expression of MEG3 and re-expression inhibits proliferation in A549 cells. (A) Relative expression of MEG3 was determined by qPCR in NHBE and A549 (p16 deletion) lung cancer cells. (B) A549 cells were transfected with either a plasmid encoding human MEG3 or empty vector and viable cell number was determined at 48, 72 and 96 h Results are shown as mean ± S.D. for results from at least three independent experiments. (C&D) Distribution of the cells in the G1, S and G2/M phases of the cell cycle after 48 h by propidium iodide (PI) staining and flow cytometry. (E&F) Apoptotic rates were measured by the presence of PI and Annexin V positive cells after 48 h by flow cytometry. *p<0.05.

To correlate the above findings to human cancer, we analyzed MEG3 expression in A549 lung adenocarcinoma cells, which harbor a p16 deletion that subsequently leads to pRb inactivation. MEG3 levels were decreased in A549 cells compared with normal human bronchial epithelial (NHBE) cells (Fig 2A). To investigate the effect of MEG3 re-expression, A549 cells were transfected with either a plasmid encoding human MEG3 or empty vector and at 48, 72 and 96 h, re-expression of MEG3 (S1 Fig) led to a significant decrease in A549 cell number (Fig 2B). To examine the effect of MEG3 on cell cycle regulation in A549 cells, cell cycle progression was analyzed by flow cytometry (Fig 2C & 2D). Cells overexpressing MEG3 showed a cell cycle arrest at G1 and had a significantly decreased G2/M phase. To determine if apoptosis also contributed to the decrease in cell number, AnnexinV/PI staining of A549 cells transfected with either a plasmid encoding mouse Gtl2 or empty vector was measure by flow cytometry (Fig 2E & 2F). Cells transfected with MEG3 showed an increase in apoptosis compared to cells transfected with empty vector. These results are consistent with the results observed for the TKO MEFS as well as with a previous study by Lu *et al*. that showed a G1 cell cycle arrest and increase in apoptosis in A549 cells after re-expression of MEG3 [3].

### Activation of pRb increases the expression of MEG3

**Fig 3:**
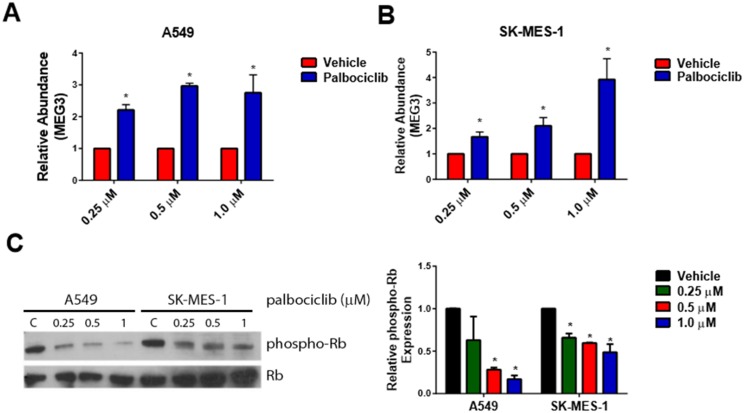
Treatment of A549 & SK-MES-1 cells with palbociclib increases expression of MEG3 in a dose-dependent manner. Relative expression of MEG3 was determined by qPCR in (A) A549 and (B) SK-MES-1 cells treated with increasing concentrations of palbociclib for 48 h. The relative abundance of MEG3 in cells treated with vehicle was set as 1. (C) Representative Western blot demonstrating phospho-Rb and total Rb in A549 and SK-MES-1 cells treated with increasing concentrations of palbociclib for 48 h. (D) Densitometry analysis of the ratio of phosphor-Rb/total Rb from A549 and SK-MES-1 cells treated with increasing concentrations of palbociclib for 48 h. Results are shown as mean ± S.D. for results from at least three independent experiments. *p<0.05.

**Fig 4:**
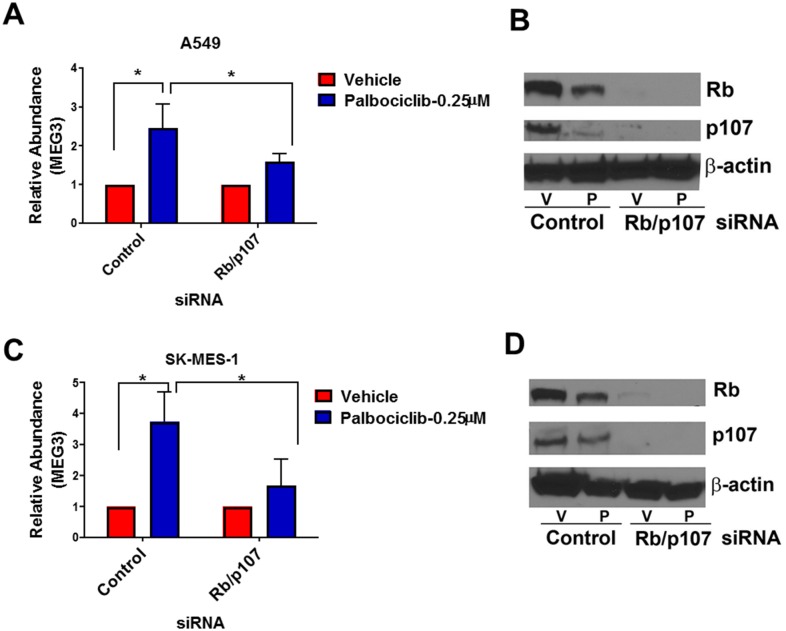
Knockdown of Rb and p107 suppresses the palbociclib-mediated induction of MEG3. Relative expression of MEG3 was determined by qPCR in (A) A549 and (C) SK-MES-1 cells transfected with either control or Rb/p107 siRNA followed by treatment with palbociclib. The relative abundance of MEG3 in cells treated with vehicle was set as 1. Knockdown of Rb and p107 were confirmed by western blot in (B) A549 and (D) SK-MES-1 cells transfected with either control or Rb/p107 siRNA followed by treatment with palbociclib (P).

**Fig 5:**
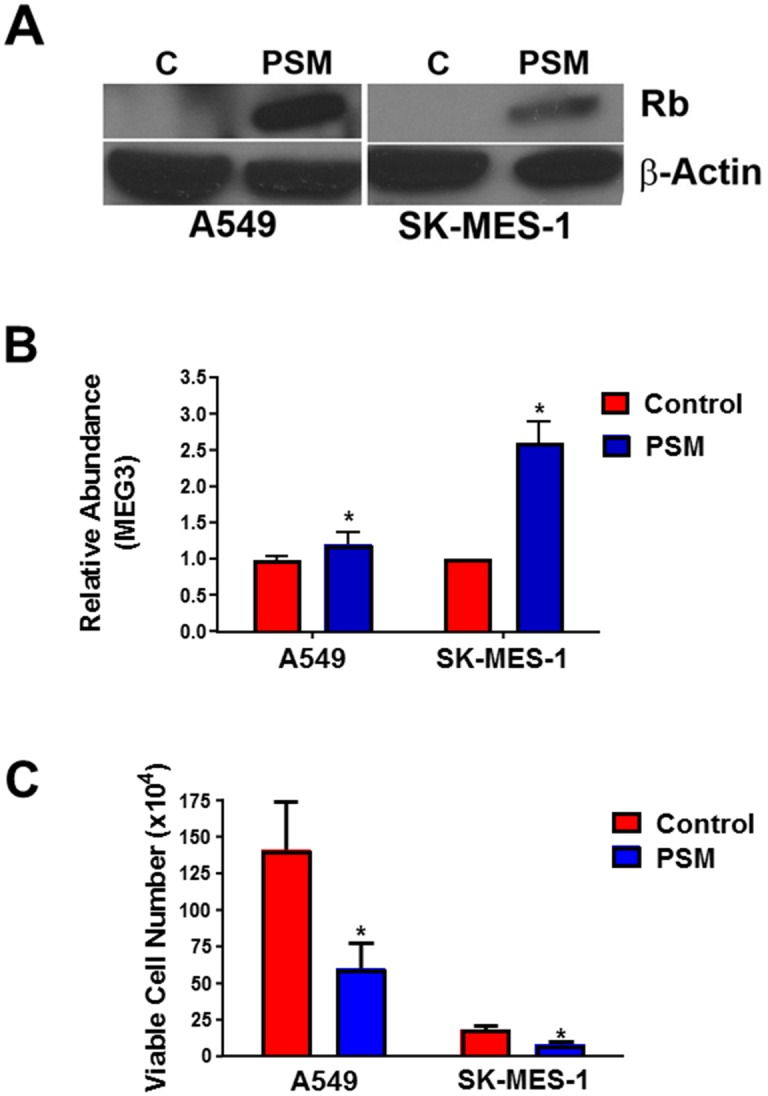
PSM-Rb increases MEG3 expression. A549 and SK-MES-1 cells were transfected with PSM-Rb or empty vector (control) for 48 h. (A) Expression of PSM-Rb was examined by western blot. (B) Relative expression of MEG3 was determined by qPCR. The relative abundance of MEG3 in cells transfected with empty vector was set as 1. (C) Viable cell number was determined.*p<0.05.

To activate the pRb pathway within the A549 and SK-MES-1 lung cancer lines, cells were treated with increasing concentrations of the CDK4/6 inhibitor, palbociclib. As measured by qPCR, expression of MEG3 increased in a dose-dependent manner following treatment with palbociclib (Fig 3A & 3B). In both the SK-MES-1 and A549 cells, Rb activation was confirmed by a decrease in phospho-Rb levels upon palbociclib treatment compared to control cells (Fig 3C & 3D). To verify that the increased MEG3 expression is mediated by pRb, cells were transfected with either a control siRNA or siRNAs to both pRb and p107 and subsequently treated with palbociclib for 48 h. Knockdown of pRb/p107 in A549 & SK-MES-1 cells significantly reduced the expression of MEG3 after treatment with palbociclib (Fig 4A & 4C). Knockdown of Rb and p107 was confirmed by qPCR (S2 Fig) and western blot (Fig 4B & 4D). To further verify that the increase in MEG3 is mediated by active Rb, A549 and SK-MES-1 cells were transfected with a constitutively active form of Rb (PSM-Rb) (Fig 5A). After transfection with PSM-Rb for 48 h, both cell lines showed an increase of MEG3 expression and a decrease in viable cell number compared to cells transfected with empty vector (Fig 5B & 5C). Together, these data indicate that MEG3 is a downstream target of the Rb pathway.

### Activation of pRb decreases expression of DNMT1 & MEG3 promoter methylation

**Fig 6:**
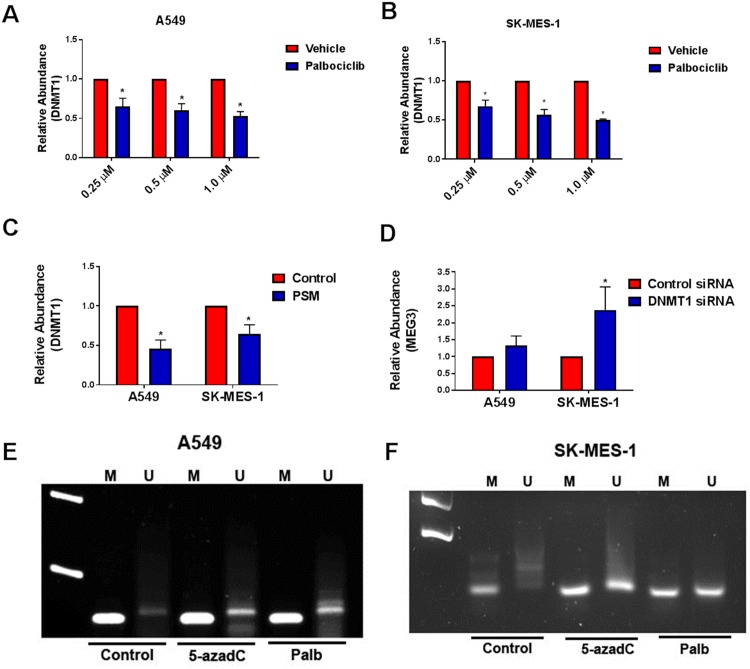
Effect of reactivation of Rb on DNMT1 expression and MEG3 promoter methylation. Relative expression of DNMT1 was determined by qPCR in (A) A549 and (B) SK-MES-1 cells treated with increasing concentrations of palbociclib for 48 h. The relative abundance of DNMT1 in cells treated with vehicle was set as 1. (C) Relative expression of DNMT1 was determined by qPCR in cells transfected with PSM-Rb or empty vector (control) for 48 h. The relative abundance of DNMT1 in cells treated with vehicle was set as 1. (D) Relative expression of MEG3 was determined by qPCR in A549 and SK-MES-1 cells transfected with either control or DNMT1 siRNA for 48 h. The relative abundance of MEG3 in cells treated with control siRNA was set as 1. *p<0.05. Results are shown as mean ± S.D. for results from at least three independent experiments. MSP analysis of MEG3 promoter methylation of (E) A549 and (F) SK-MES-1 cells treated with 5-azadC or palbociclib for 48 h. Representative results of PCR products of methylated (M) and unmethylated (U) alleles are shown for three independent experiments.

We next investigated the mechanism by which the Rb pathway may control MEG expression. Since pRb is known to transcriptionally regulate DNMT1, we determined the effect of palbociclib mediated pRb activation on DNMT1 expression levels. When A549 and SK-MES-1 cells were treated with increasing concentrations of palbociclib, we found that expression of DNMT1 decreased in a dose-dependent manner following treatment with palbociclib (Fig 6A & 6B). Transfection with the constitutively active PSM-Rb also resulted in a decrease in DNMT1 levels after 48 h compared to cells transfected with empty vector (Fig 6C). Since DNMT1 has previously been shown to regulate MEG3 expression through methylation in human hepatocellular carcinoma and hepatic stellate cells [14, 15], we next wanted to determine the effect of DNMT1 knockdown on MEG3 levels within these lung cancer cell models. A549 and SK-MES-1 cells were transfected with either control or DNMT1 specific siRNA (S3 Fig) and silencing of DNMT1 resulted in increased expression of MEG3 in both cell types (Fig 6D). To determine if activation of Rb affects MEG3 promoter methylation, we performed methylation-specific PCR on cells treated with the methylation inhibitor 5-azadC or with palbociclib. In both cell types, treatment with either 5-azadC or with palbociclib resulted in an increase in the unmethylated state of MEG3 (Fig 6E & 6F). Together, these results demonstrate that Rb activation leads to decreased DNMT1 and decreased methylation of the MEG3 locus.

### Silencing of MEG3 partially rescues the palbociclib-mediated decrease in cell proliferation

**Fig 7:**
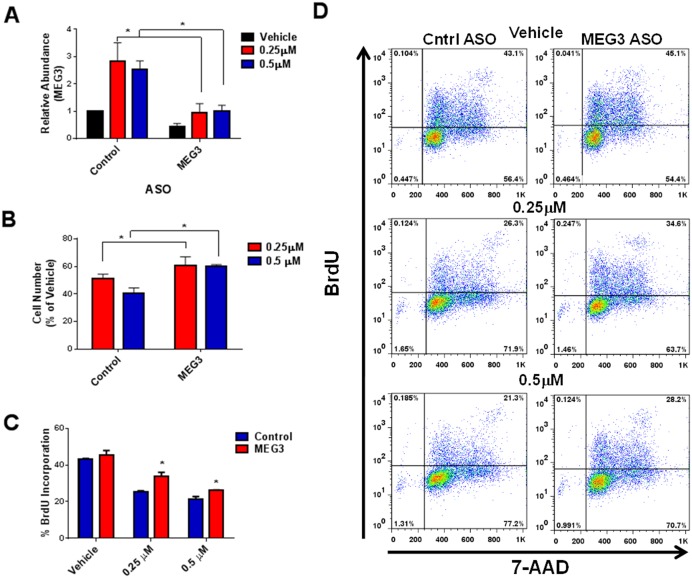
ASO treatment suppresses the palbociclib-mediated induction of MEG3 and decrease in cell proliferation in A549 cells. (A) Relative expression of MEG3 was determined by qPCR in A549 cells treated with control or MEG3 ASO for 24 h followed by treatment with 0.25 μM or 0.5 μM palbociclib for 48 h. The relative abundance of MEG3 in control ASO cells treated with vehicle was set as 1. Results are shown as mean ± S.D. for results from at least three independent experiments. (B) Viable cell number was calculated as percent of vehicle control. Results are shown as mean ± S.D. for results from at least three independent experiments. (C) BrdU incorporation was measured in cells treated with control or MEG3 ASO for 24 h followed by treatment with 0.25 μM or 0.5 μM palbociclib for 48 h. (D) Representative flow plots showing BrdU incorporation.

**Fig 8:**
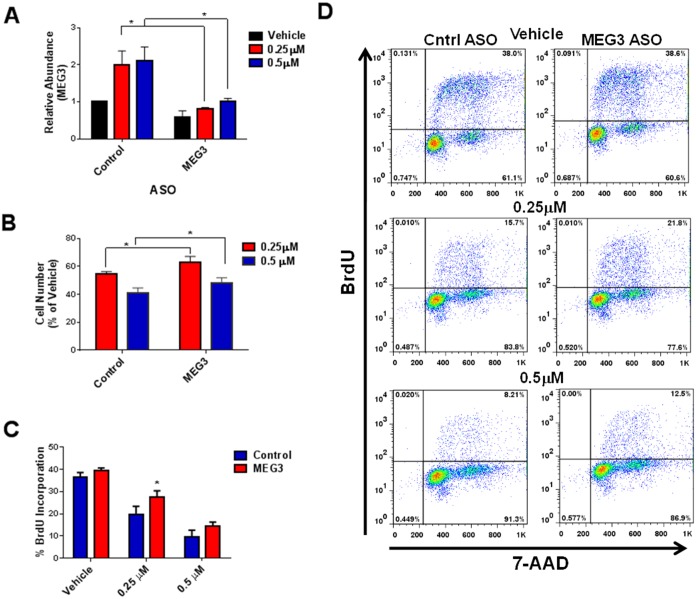
ASO treatment suppresses the palbociclib-mediated induction of MEG3 and decrease in cell proliferation in SK-MES-1 cells. (A) Relative expression of MEG3 was determined by qPCR in SK-MES-1 cells treated with control or MEG3 ASO for 24 h followed by treatment with 0.25 μM or 0.5 μM palbociclib for 48 h. The relative abundance of MEG3 in control ASO cells treated with vehicle was set as 1. Results are shown as mean ± S.D. for results from at least three independent experiments. (B) Viable cell number was calculated as percent of vehicle control. Results are shown as mean ± S.D. for results from at least three independent experiments. (C) BrdU incorporation was measured in cells treated with control or MEG3 ASO for 24 h followed by treatment with 0.25 μM or 0.5 μM palbociclib for 48 h. (D) Representative flow plots showing BrdU incorporation.

Next, we wanted to determine whether silencing the palbociclib-mediated increase in MEG3 could rescue the drug induced decrease in cell proliferation. A549 and SK-MES-1 cells were transfected with control or MEG3 ASO for 24 h followed by treatment palbociclib. MEG3 expression levels were significantly decreased in cells treated with the MEG3 specific ASO compared to control after treatment with palbociclib in both cell types (Figs 7A & 8A). Loss of MEG3 expression partially rescued the palbociclib-mediated decrease in cell number (Figs 7B & 8B). Correspondingly, loss of MEG3 expression also resulted in increased cell proliferation measured by BrdU incorporation in palbociclib treated cells (Figs 7C, 7D, 8C and 8D). Combined, these studies suggest that MEG3 induction contribute, in part, to the decrease in cell proliferation upon Rb pathway activation.

### MEG3 expression is lower in clinical lung adenocarcinomas with RB pathway inactivation

**Fig 9:**
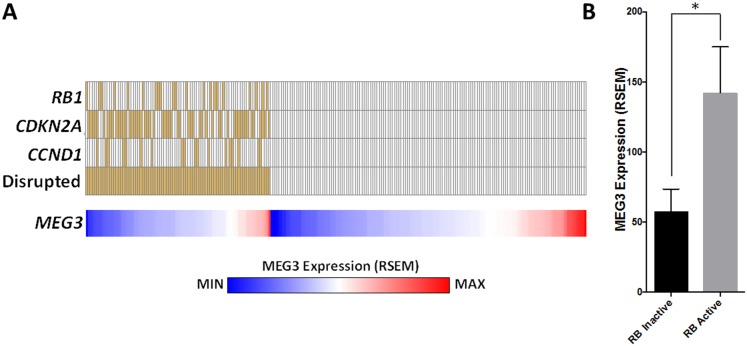
MEG3 expression is lower in clinical lung adenocarcinomas with RB pathway inactivation. Genomic data from the lung adenocarcinoma TCGA dataset was downloaded from the cBIO portal (n = 230). The RB pathway was considered disrupted in a tumor if one of the following events occurred: 1) RB1 mutation/homozygous deletion/underexpression; 2) CDKN2A mutation/homozygous deletion/underexpression; or 3) CCND1 overexpression. Tumors without any of these events were considered to have an active RB pathway. *MEG3* expression was then plotted for each group (A) and compared between the groups (B). *p≤0.01, Unpaired t test with Welch’s correction.

Finally, we wanted to determine if there is a relationship between MEG3 and Rb pathway disruption in human lung tumors. Genomic data from the lung adenocarcinoma TCGA dataset was downloaded from the cBIO portal. The RB pathway was considered disrupted in a tumor if one of the following events occurred: 1) RB1 mutation/homozygous deletion/underexpression; 2) CDKN2A mutation/homozygous deletion/underexpression; or 3) CCND1 overexpression. Tumors without any of these events were considered to have an active RB pathway. *MEG3* expression was then plotted for each group (Fig 9A) and compared between the groups (Fig 9B). The results demonstrate that MEG3 expression levels are significantly decreased in tumors with a disrupted Rb pathway.

## Discussion

The pRb pathway plays a critical role in cell cycle progression and is disrupted in many human cancers. In the absence of a mitogenic or oncogenic signal, hypophosphorylated pRb forms a complex with the E2F family of transcription factors, which blocks the expression of several cell cycle regulatory genes causing cell cycle arrest. Genetic deletion, mutation or inactivation of pRb prevents the E2F complexes from forming, thereby promoting cell cycle progression and proliferation [20]. Although this canonical function of pRb has been well characterized, recent studies have revealed that pRb plays a role in the regulation of additional cellular processes, such as apoptosis, differentiation, and epithelial-to-mesenchymal transition [21–23]. In addition, both E2F dependent and independent mechanisms are being characterized that describe how pRb can control such a variety of cellular processes.

Our studies have identified a novel mechanism by which pRb controls cellular proliferation via increased expression of the lncRNA MEG3. Here, we have shown that activation of pRb causes decreased expression of DNMT1, allowing for increased MEG3 expression and decreased cancer cell growth. Activation of pRb by treatment with palbociclib or introduction of a constitutively active form of Rb increased the expression of MEG3 in a dose-dependent manner. Introduction of a constitutively active PSM-Rb also led to an increase in MEG3 expression. Notably, the palbociclib mediated decrease in MEG3 was attenuated by knockdown of pRb/p107, showing that this increased MEG3 expression is mediated through Rb pathway activation. Treatment with palbociclib and transfection of PSM-Rb decreased the expression of DNMT1, while, conversely, knockdown of DNMT1 resulted in increased expression of MEG3, indicating that DNMT1 contributes to the silencing of MEG3. This is consistent with results from He *et al*. that showed that MEG3 expression was increased with DNMT1 knockdown in human hepatic stellate cells [15]. In further support of methylation as a mechanism for Rb mediated regulation of MEG3, treatment with palbociclib also led to a change in methylation of the MEG3 promoter. Silencing of drug-induced MEG3 expression partially rescued the palbociclib-mediated decrease in cell proliferation. Finally, we found a correlation between Rb pathway disruption and MEG3 levels in human lung tumors, and taken together, these data suggest that disruption of the pRb-DNMT1 pathway leads to a decrease in MEG3 expression, thereby contributing to the pro-proliferative state of certain cancer cells.

The mechanisms by which MEG3 inhibits cell proliferation are still under investigation. Zhou *et al*. initially characterized MEG3 as an activator of p53 and showed that MEG3 up-regulates the expression of the p53 target gene GDF15 [7]. Interestingly, this study also showed that MEG3 inhibited cell proliferation in a p53-independent manner, indicating that MEG3 functions through an additional pathway. Lu *et al*. also showed that MEG3 inhibits cell proliferation at least partially by the activation of p53 [3]. A more recent study by Mondal *et al*. showed that MEG3 specifically regulates the TGF-β signaling pathway by targeting the chromatin through the formation of RNA-DNA triplex structures [8]. These triplex structures may represent a general mechanism by which MEG3 can interact with chromatin and affect the expression of a variety of target genes. These studies provide an additional link between pRb and p53 regulation and many downstream pathways, such as the TGF-β pathway.

The expression of MEG3 has been shown to be altered in many human cancers, such as non-small cell lung cancer (NSCLC), hepatocellular carcinoma, meningiomas, gastric cancer and pituitary tumors [3, 12, 14, 24, 25]. Therefore, elucidating the mechanisms by which MEG3 expression can be restored will give insight into potential new cancer therapeutics. In this study, we show that treatment with palbociclib, a CDK4/6 inhibitor currently used in the treatment of metastatic breast cancer, increased the expression of MEG3 in the lung cancer cell lines A549 and SK-MES-1. This suggests that palbociclib or other CDK4/6 inhibitors could be used therapeutically to target multiple tumor types that rely on MEG3 regulated pathways for growth. Additionally, these inhibitors could potentially be used in combination with other agents that are currently in clinical use. For example, decreased MEG3 expression has been correlated with cisplatin resistance in NSCLC, and re-expression of MEG3 in cisplatin resistant A549/DDP cells increased the sensitivity to this drug [26]. Patients with NSCLC are commonly treated with cisplatin-based chemotherapies; however acquired resistance to this drug often limits its success. Our results suggest a potential therapeutic utility for CDK4/6 inhibitors in NSCLC, the mechanism of which is related to increased MEG3 expression. In addition to lung cancer, increasing MEG3 expression may also provide a therapeutic benefit in other tumor types. Palbociclib prolongs progression-free survival in advanced stage breast cancer and has been recently approved by the FDA for use in these patients [27]. TGF-β signaling has also been shown to have a major role in breast cancer metastasis, and inhibitors of this pathway are currently undergoing testing in multiple clinical trials [28–30]. Since MEG3 has recently been shown to regulate the TGF-β signaling pathway in breast cancer cells [8], our data indicate that the combinatorial administration of palbociclib with TGF-β inhibitors (e.g. galunisertib) may be of therapeutic benefit to breast cancer patients. Together, since MEG3 potentially regulates multiple downstream pathways, Rb mediated induction of MEG3 may not only be important in the treatment of NSCLC and breast cancer, but in other cancer types that are responsive to palbociclib, and further studies are needed to determine the potential benefit of combinatorial therapies against these cancers.

## Supporting Information

S1 Fig Expression of Gtl2/MEG3 in TKO MEF cells or A549 lung cancer cells. (A) Relative expression of Gtl2 was determined by qPCR in MEFs transfected with either a plasmid encoding mouse Gtl2 or empty vector. (B) Relative expression of MEG3 was determined by qPCR in A549 cells transfected with either a plasmid encoding human MEG3 or empty vector at 48 h.
(TIF)

S2 Fig Confirmation of Rb and p107 knock-down in lung cancer cells by qPCR. Relative expression of Rb or p107 was determined by qPCR in (A) A549 and (B) SK-MES-1 cells transfected with either control or Rb/p107 siRNA for 48 h. *p<0.05. The relative abundance of Rb and p107 in cells treated with control siRNA was set as 1. Results are shown as mean ± S.D. for results from at least three independent experiments.
(TIF)

S3 Fig Confirmation of DNMT1 knock-down in lung cancer cells by qPCR. Relative expression of DNMT1 was determined by qPCR in (A) A549 and (B) SK-MES-1 cells transfected with either control or DNMT1 siRNA for 48 h. *p<0.05. The relative abundance of DNMT1 in cells treated with control siRNA was set as 1. Results are shown as mean ± S.D. for results from at least three independent experiments.
(TIF)
