# Supplementary figures and images for: Expression of the lncRNA Maternally Expressed Gene 3 (MEG3) Contributes to the Control of Lung Cancer Cell Proliferation by the Rb Pathway
Source: PLoS One. 2016 Nov 10;11(11):e0166363. doi: 10.1371/journal.pone.0166363 (PMC5104461; doi:10.1371/journal.pone.0166363)

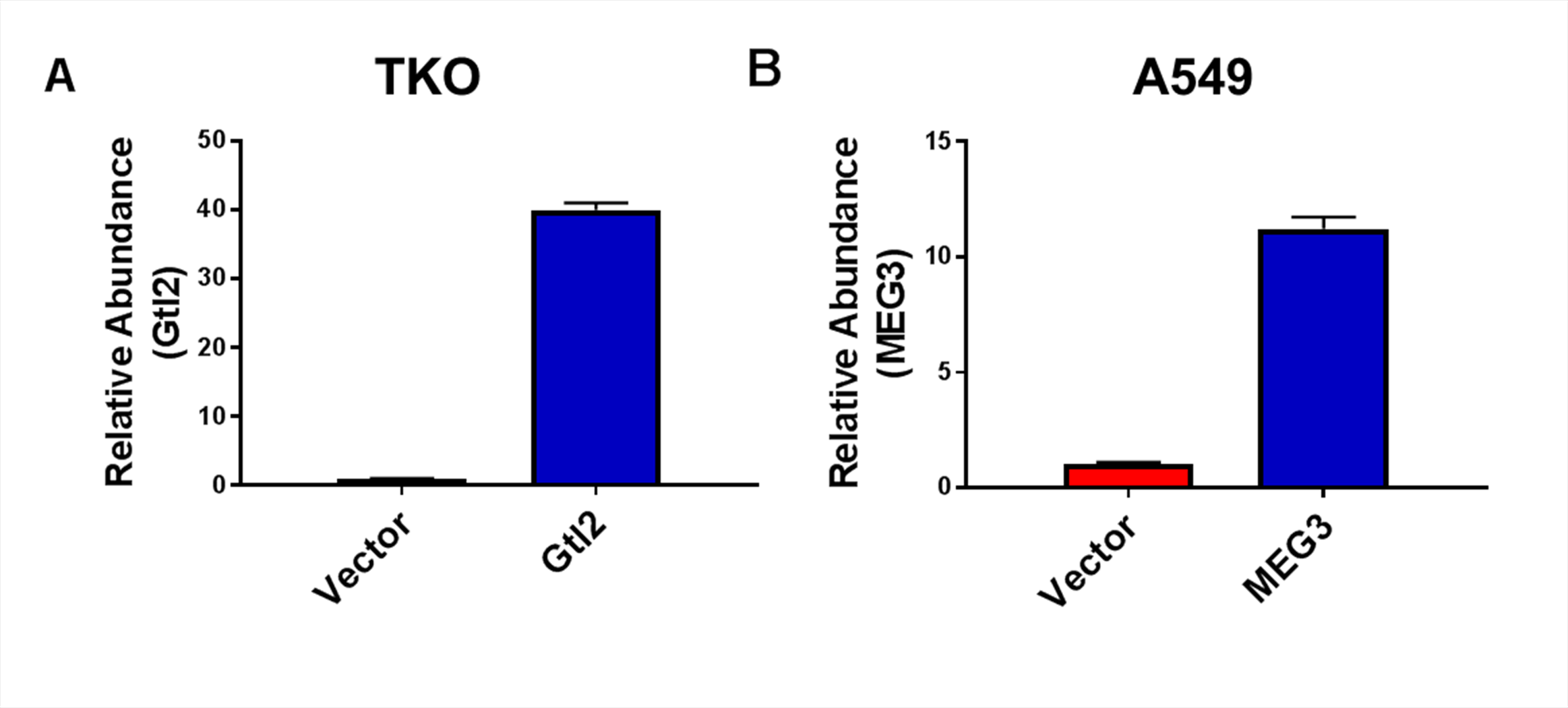

Supplement: S1 Fig — (A) Relative expression of Gtl2 was determined by qPCR in MEFs transfected with either a plasmid encoding mouse Gtl2 or empty vector. (B) Relative expression of MEG3 was determined by qPCR in A549 cells transfected with either a plasmid encoding human MEG3 or empty vector at 48 h. (TIF) [file pone.0166363.s001.tif]

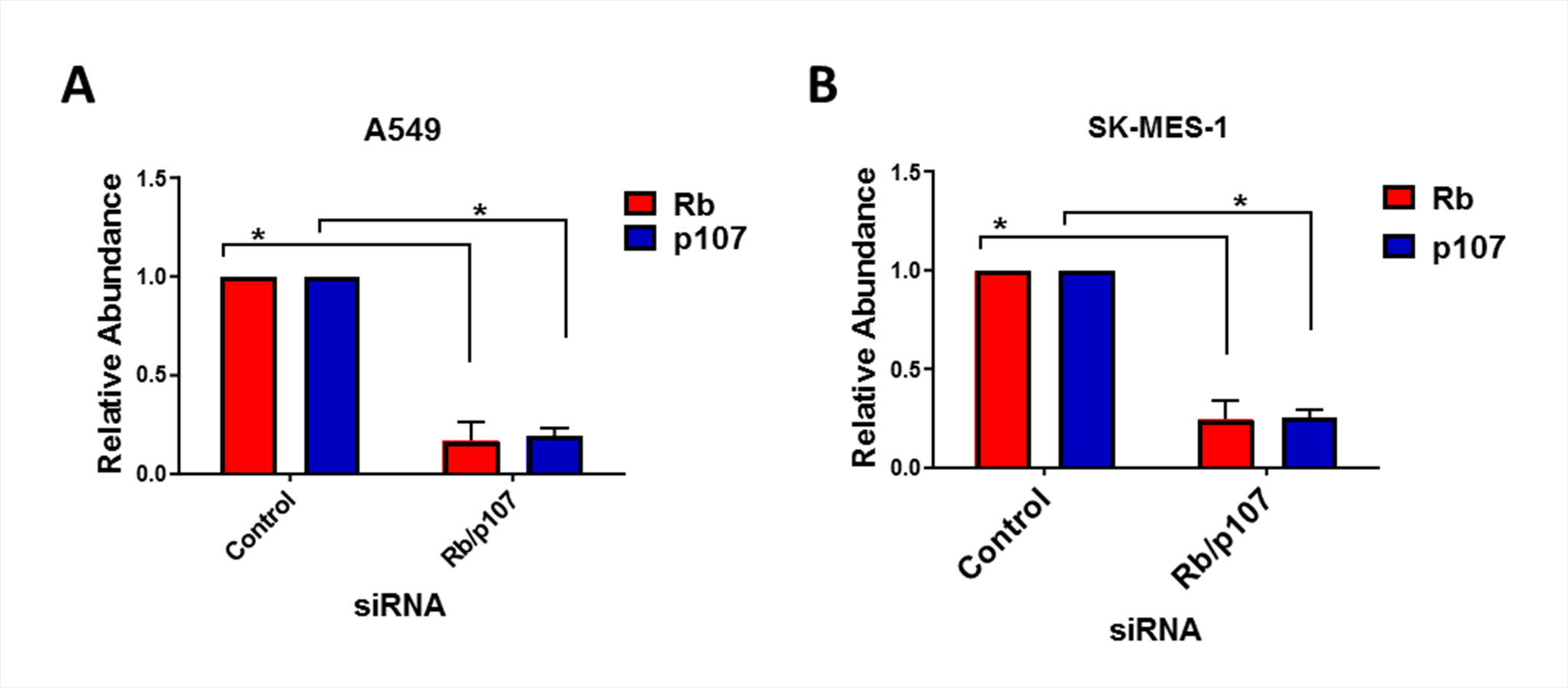

Supplement: S2 Fig — Relative expression of Rb or p107 was determined by qPCR in (A) A549 and (B) SK-MES-1 cells transfected with either control or Rb/p107 siRNA for 48 h. *p<0.05. The relative abundance of Rb and p107 in cells treated with control siRNA was set as 1. Results are shown as mean ± S.D. for results from at least three independent experiments. (TIF) [file pone.0166363.s002.tif]

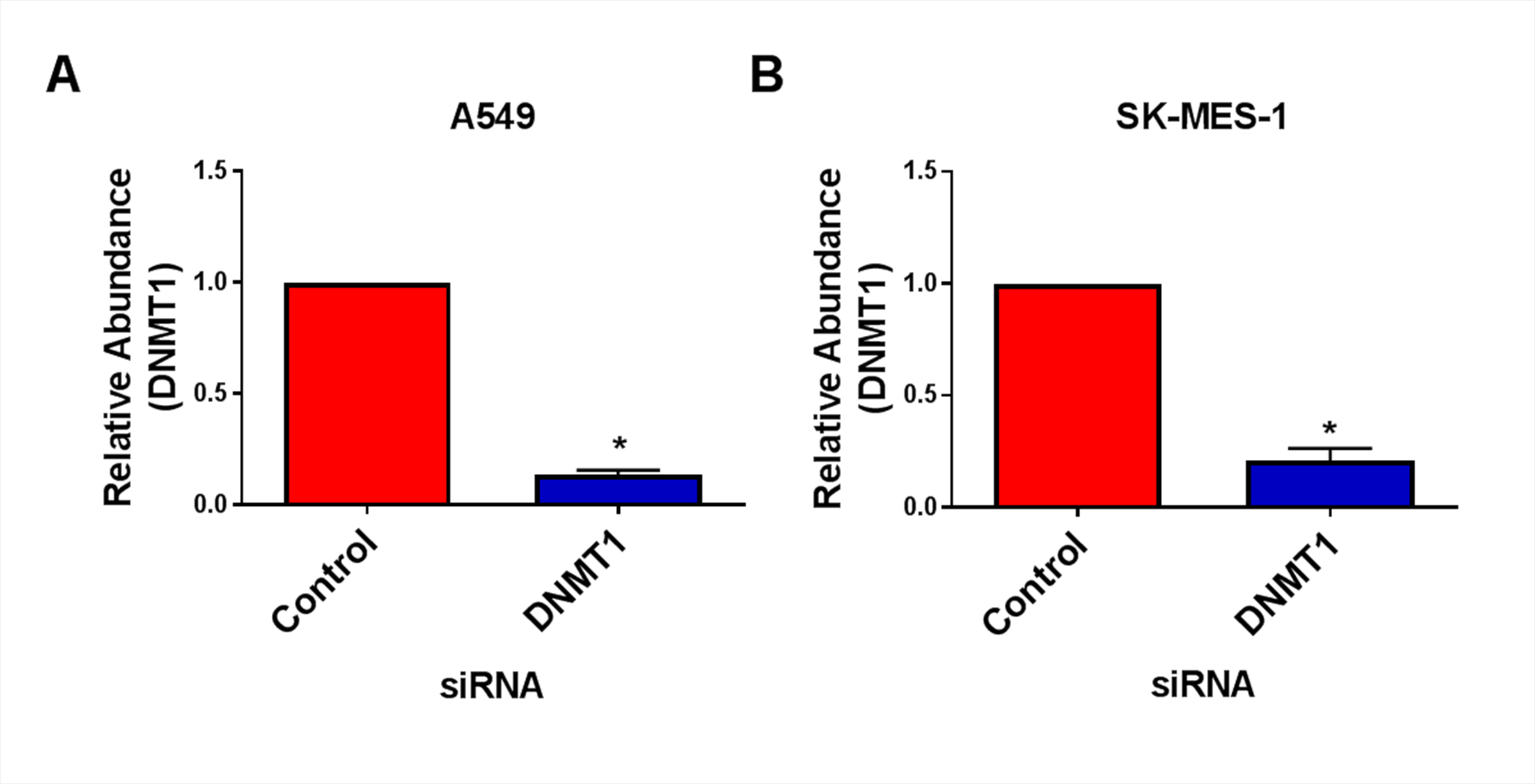

Supplement: S3 Fig — Relative expression of DNMT1 was determined by qPCR in (A) A549 and (B) SK-MES-1 cells transfected with either control or DNMT1 siRNA for 48 h. *p<0.05. The relative abundance of DNMT1 in cells treated with control siRNA was set as 1. Results are shown as mean ± S.D. for results from at least three independent experiments. (TIF) [file pone.0166363.s003.tif]
